# Health literacy education at the time of COVID-19: development and piloting of an educational programme for university health professional students in 4 European countries

**DOI:** 10.1186/s12909-023-04608-3

**Published:** 2023-09-08

**Authors:** Roberta Papa, Jane Sixsmith, Cinzia Giammarchi, Sonia Lippke, Verna McKenna, Lucia Di Furia, Maria Gabriella Ceravolo, Andrea De Winter

**Affiliations:** 1 Regional Health Agency Marche Region, Palazzo Rossini - via Gentile da Fabriano n.3, Ancona, 60125 Italy; 2 IRCCS INRCA, Ancona, Italy; 3 Health Promotion Research Centre, Discipline of Health Promotion, National University of Ireland Galway, Galway, Ireland; 4 Constructor University, Bremen, Germany; 5 Department of Experimental and Clinical Medicine, Polytechnic University of Marche, Ancona, Italy; 6 Department of Health Sciences, University Medical Centre Groningen, University of Groningen, Groningen, The Netherlands

**Keywords:** Health literacy, Higher education, Healthcare professionals, COVID-19, Patient centred-care, Curriculum, Online training

## Abstract

**Background:**

Health literacy has a strong influence on individual health outcomes and the sustainability of healthcare systems. Healthcare professionals often overestimate patients’ health literacy levels and lack adequate competencies to address limited health literacy effectively. Therefore, promoting understanding through effective health communication between professionals and citizens is becoming increasingly important. Although health literacy has recently gained more attention, health literacy educational programmes targeting future healthcare professionals are still scarce, especially in Europe. This study describes the piloting process of a pan-European health literacy educational programme and shows how the educational material is being used during time of crisis such as the COVID-19 pandemic.

**Methods:**

The educational programme was developed through the definition of an educational philosophy and iterative co-creation processes consisting of stakeholders’ consultations, material development and pilots with students. The evaluation was carried out in Italy through four pilot tests involving 107 students of health-related degrees. An evaluation questionnaire and a pre-post test were developed and used to collect students’ and educators’ feedback (quantitative and qualitative) and assess changes in health literacy awareness, respectively. Three additional pilots were organized in Italy and Germany mostly during the COVID-19 pandemic to evaluate the feasibility of the educational programme through online and hybrid learning, respectively.

**Results:**

The pilots received positive feedback from both students and educators. Students were highly satisfied with the courses, reported their relevance for their future profession and appreciated the interactive teaching methods. The pre-post test showed a significant improvement in health literacy awareness after the training. Educators reported the adequacy and flexibility of the training material, the ease of transferability of the content of the lessons into practice, and the validity of the tested options to integrate the educational programme into the curricula.

**Conclusions:**

Our comprehensive, evidence-based educational programme contributes to addressing the existing challenges in Europe, and its flexibility allows for easy integration in the curricula, through different options, hence supporting a widespread uptake in the European Union and maybe beyond. Health literacy education is a useful tool to improve citizens’ access to healthcare information and services, achieve better health outcomes and support healthcare systems’ sustainability.

**Supplementary Information:**

The online version contains supplementary material available at 10.1186/s12909-023-04608-3.

## Background

Health literacy (HL) is the ability to access, understand, appraise and communicate health-related information [1, 2]. HL has a strong impact on health at both individual and societal levels [3]. Indeed, individuals with limited HL – estimated as 47% of Europeans [4] - are more likely to face difficulties in accessing and making use of healthcare services, resulting in worse health outcomes, higher costs for the healthcare system, and increased health inequalities [5–9] [10]. People with limited HL benefit specifically from person-centred care, health promotion, and interventions to promote well-being and safety. The COVID-19 pandemic has highlighted even more the impact of limited HL, showing both how this problem is globally underestimated [11, 12] and its huge impact, the so-called ‘infodemic’, where people struggled to navigate, understand, appraise and use appropriately the mass of information, including misinformation provided by media [13].

To effectively address HL and thus enhance the sustainability of healthcare systems [14], a comprehensive approach is required, that can target simultaneously citizens, communities, professionals, and healthcare organizations [15, 16]. To achieve this goal, a dynamic, resilient workforce - equipped with appropriate competences to work in multidisciplinary teams and address the complex care needs of the population [17–19] - is needed, together with evidence-based skills set with complementary health system structures [20, 21]. Co-creation processes in developing and improving them iteratively is key as it was also found in the past (e.g. [22]).

Healthcare professionals often overestimate patients’ HL levels and lack adequate competencies to address limited HL effectively [23–25]. Although HL education has recently gained more attention, HL educational programmes targeting future healthcare professionals are still scarce or address a limited set of health literacy competences. Health Literacy education is most evident in Australia and the US [26, 27], with the latter having included HL education in the 63% of schools providing bachelor programmes for nurses [28]. In Europe, health literacy courses were developed and tested in higher education settings in Germany [16] and Spain [29]. Furthermore, a randomized controlled trial was carried out in The Netherlands, and was found to be effective in increasing the health literacy competencies of undergraduate medical students [30]. These studies found that, after training, future healthcare professionals showed positive behaviour changes, were more aware of the needs of patients with limited HL, and more skilled in providing comprehensible information, enabling effective shared decision-making and promoting patient self-management [30–34].

Based on this context, the objective of the IMPACCT project (IMproving PAtient-centered Communication Competences: To build professional capacity concerning health literacy in medical and nursing education) is to strengthen a broad set of professional health literacy competences, by developing and testing an evidence-based educational programme on health literacy for healthcare students in Europe by means of traditional and new learning approaches [22, 35].

The purpose of this paper is to describe the three main phases of the study, with a focus on the third one, i.e. the pilots of the HL educational programme and their evaluation process among students and educators. Moreover, it shows how the educational material is being used by educators prior and during the COVID-19 pandemic. This phased approach provides the structure for the reporting of this study.

## Methods

### Study design

The 3 main phases of the study were: (1) the definition of the programme framework and educational philosophy; (2) the development of the educational programme through an iterative co-creation process in 3 European countries (The Netherlands, Germany, Ireland); (3) the piloting of the educational programme in a real-world scenario, i.e. a higher education setting, in Italy.

In addition, prior and during the COVID-19 pandemic, the training material was tested through online or hybrid teaching/learning in two countries (Italy, Germany). These pilots are reported here as examples of the use of the educational material in the framework of hybrid teaching/learning scenario.

We used mixed methods with a maximum variation sampling strategy [36, 37], to reflect in the programme development and evaluation the diversity in health and education systems of the participating countries and organizations and, at the same time, identify shared patterns that emerge from the heterogeneity across the settings.

**Fig. 1:**
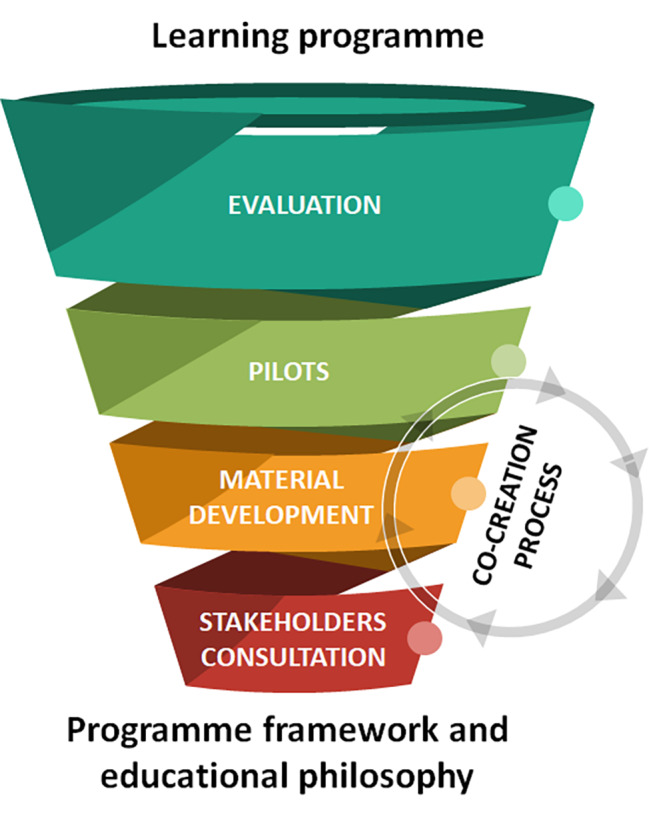
The development process of the educational programme

An iterative process (Fig. 1) was applied, to continuously revise the educational material to incorporate perspectives and needs of educators, students, and other relevant stakeholders (e.g., healthcare professionals, patients, etc.). The study was carried out in accordance with relevant guidelines and regulations, and approved by the respective ethical committees.

### Phase 1 - Programme framework and educational philosophy

The first phase of the study included the development of an educational philosophy based on educational theories aligned with the Bologna Process [38], which started in 1999 to ensure that higher education systems in Europe are coherent, comparable and compatible, fostering European co-operation and quality assurance in higher education - to enable programme transnational transferability. Moreover, a systematic literature review was carried out to formulate learning outcomes.

The programme was founded on student-centred and competency-based education. Student-centred education refers to active and engaged students with autonomy and responsibility for their learning [39–41] with the function of programme content and delivery to contribute to the learning process and skills acquisition [42]. Competency-based education is about learning related to not just knowledge acquisition but also skills, as well as concepts such as attitudes, values and beliefs [43]. Bigg’s model of constructive alignment [35] underpinned the development process with learning outcomes, as defined by the Bologna Working Group [44], reflected in curriculum content and assessment.

The learning outcomes and subject content were formulated into learning units (LUs) comprising sets of teaching materials in a specific HL subject area. Structure and content were informed by the needs, experiences and preferences of patients and by a systematic review of qualitative and mixed methods studies [45]. This review reports the details of the needs, experiences and preferences of patients with limited HL and chronic diseases, the set of learning outcomes and the person-centred educational framework developed. Four main themes were derived from the selected articles representing aspects evaluated by patients as relevant to their care process (i.e., support system; patient self-management; healthcare professionals’ interpersonal capacities; barriers in the healthcare system), and two transversal recurrent themes (i.e., cultural sensitivity; eHealth). These themes were used to formulate specific learning outcomes that would be truly person-centred, relevant to patients, and helpful to prioritize what future healthcare providers should learn.

### Phase 2 - co-creation to develop the educational programme

The development process comprised an iterative co-creation process consisting of stakeholders’ consultations, material development and co-creation pilots with students.

#### Consultations with stakeholders

There were three phases of stakeholder co-creation activities, which were organized during the project’s transnational meetings through participatory stakeholders’ workshops. Stakeholders (e.g. educators, students, patients, policy makers) were selected on the basis of their expertise comprising, knowledge, skills, experience and insights, aligning with the projects aims and objectives. They were invited through a trailer, press releases, other meetings and e-mails. Each phase allowed the design of the educational programme to be aligned with the perspectives and needs of healthcare professionals, patient organisations, and students. The main suggestions received and adopted were: inclusion of specific topics of interest such as identification of health literacy problems, patient-provider interaction barriers, and patients’ preferences (e.g. culture, education, experiences); mental health literacy; flexibility of the programme to different settings (the “supermarket model”); promotion of the programme to key stakeholders. These inputs were also considered in the design and evaluation of the pilots. The summary of outcomes of the consultations and consequences for the implementation are described in more detail [see Additional file 1].

#### Material development

A document template for the initial development of learning units was constructed from the educational and philosophical framework [16]. This included: background information and current research, learning outcomes, level of the materials (basic or advanced), target audience, type of materials (e.g. lectures, group work, role play, discussion and debates), and assessments.

Each project partner was tasked with learning units to develop, allocated through discussion and based on expertise, teaching experience and interest. On completion, the documents were peer-reviewed by a project partner and feedback was provided, which was incorporated by the learning unit development. This provided a foundation and pathway from which coherent, consistent content and materials across the programme were developed.

The consistency and coherence of development across the programme were facilitated through a navigation template [see Additional file 2. S. Table 1] that documented student learning outcomes, with educational activities [see Additional file 2. S. Table 2], teaching and learning materials, and assessment. Finally, all learning materials comprising the educational programme [see Additional file 2. S. Table 3] were incorporated into a manual for educators.

#### Co-creation pilots with students

Co-creation pilots with students were implemented in Ireland, Germany, and the Netherlands. They included the development and testing of parts of the training material, with feedback used to refine the educational programme. A mixed-methods approach was used to pilot test nine learning units of the educational programme. The students’ feedback was collected through rating scales, verbal feedback rounds, group interviews and observations during the activities. Results are shown in Table 1. In general, educators and students perceived the learning units tested as relevant, useful and feasible. Participants provided valuable suggestions to improve the content or strategies to tailor the learning units more to the needs of students or educators. The findings [46] were incorporated into the programme.

**Table 1:** Co-creation pilots with students: results and changes made

Country	Co-creation activity	Target group	Pilots (n)	Overall participants (n)	Feedback provided	Actions implemented
Ireland	Group Interview	Masters Students	1	12	- More experiential learning methods and real-world examples - More guidance for educators and detail for role play - Re-evaluate the balance between didactic and experiential teaching	- Guidance on how to choose and use materials in the manual - Wider range of real-world examples developed - More emphasis placed on experiential teaching
	Education – module	Bachelor students	1	5		
	Education-Lecture and Group work	Bachelor students	3	205		
Germany	Training	Bachelor students	6	593	- Overall positive feedback - Special interest in easy-to-implement/low level movement interventions into everyday life - Students not familiar with reading research articles - More explanations - More examples for application and implementation of behaviour change techniques	- More tests to check students’ understanding of the content - More practical exercises and patient cases - More individual activities - Add an agenda and all sources and further readings - Match lessons to the skill level of the audience - Get practitioners from different disciplines into the class to talk about their jobs and suggestions
	Seminar	Bachelor students	4	45		
	Online seminar	Bachelor students, different health-related degrees	1	31		
	Seminar	Master students	2	19		
	Lecture	Bachelor and Master’s students	2	84		
	Workshop	Trainers, administrators and multipliers	1	25		
Netherlands	Training	Bachelor students	3	33	- More cases and role-plays - Clear instructions on assessment - Including practical examples (e.g. videos) - Improving relevance to students with any background/expertise - Including small group activities to share experiences - Let a patient or medical specialist tell their personal story. - Trainer playing the patient during role-play	- More practical examples and exercises - Improved instructions - Improved content flexibility - Suggestion to have patients/medical specialists in the class
Total			24	1052		

### Phase 3 - piloting of the educational programme

The educational programme was tested in Italy through four pilot tests, namely Pilots 1–4. As this country was not directly involved in the development process, the pilots allowed for ‘validation’ of the programme by evaluating its feasibility and transferability in other educational settings, as well as testing its relevance and ease of integration into the curricula through different scenarios.

#### Context

The pilots were carried out from November 2018 to October 2019 at the Faculty of Medicine and Surgery of the Polytechnic University of Marche (UNIVPM), in the Marche region. The University offers 14 undergraduate courses, 40 post-graduate courses, a PhD programme and a Masters’ course in Narrative Medicine, Communication and Ethics of Care.

#### Procedures

The project team presented the educational programme to the Faculty Dean and staff. Those interested in participating in the project selected the learning units (LU) to be tested, taking into account the objectives of the courses, the time available for training and the skill level of the students. This activity resulted in the definition of possible ways (i.e. pilots) to integrate the educational programme in the existent curriculum according to the structure of the educational activities already in place. Then, they revised the training material, making small adaptations to contextualize it. The project team supported the educators in the translation of the material (when needed), participated as auditors in the training, and collaborated in the evaluation.

#### Description of pilot features

Pilot characteristics are summarized in Table 2. They were carried out in different periods of the year allowing to use results and inputs of the educators and students to improve the procedures of subsequent pilots (e.g. selection of the educational material). Two pilots were arranged as optional thematic courses, i.e. training activities specifically dedicated to a subject, which the students were able to choose as part of a wider training proposal. Pilot 1 was jointly organised and mostly oriented towards advanced skills and promoted collaboration and exchange between the students. Pilot 2 (online) integrated HL (4 modules) and healthcare communication (4 modules) into the topic of pain therapy and palliative care in nervous system diseases (5 modules). Pilot 3 was organized by adding 3 lessons to the regular compulsory training to provide basic information on HL and address the cross-cutting issue of diversity. Finally, in Pilot 4 the educational activities were proposed as optional additional activities which students, who were at an advanced stage of their studies, could choose. The lessons aimed to provide a short introduction to HL and a focus on specific aspects of the midwifery profession, such as the interaction with patients from different cultural backgrounds.

**Table 2:** Piloting of the educational programme: pilot test characteristics

Features	Pilot 1	Pilot 2	Pilot 3	Pilot 4
*Course type*	Thematic course (optional)	Thematic online course (optional)	Part of a Laboratory (mandatory)	Optional extra activity
*Target group*	Third-year students, Nursing and Speech Therapy courses	Fifth-year students, Medicine and Surgery course	First-year students, Physiotherapy course	Second and third-year students, Midwifery course
*Duration*	3 lessons (including evaluation), 9 h	8 modules, 10 h	3 lessons^a^, 7.5 h, followed by evaluation	3 lessons (including evaluation), 9 h
*Learning units tested*	HL Canon, Diversity, Increasing participation during the consultation, Adherence, Improving health behaviours	HL Canon, Diversity, Increasing participation during the consultation	HL Canon, Diversity	HL Canon, Diversity, Identification of HL Problems
*Adaptations to the material*	Partial translation, summary to fit the time available	Partial translation, summary to fit the time available	Partial translation, summary to fit the time available, discussions in pairs instead of groups for some activities	Partial translation, summary to fit the time available, a focus on patients with different cultural backgrounds.
*Additional material used*	Specific tools and questionnaires; Italian statistical data on HL; videos in Italian; real-time word-cloud followed by group discussions during each meeting; stories for role-play	Specific tools and questionnaires; Italian statistical data on HL; video in Italian	Italian statistical data on HL; videos in Italian; a specific assignment related to the internship	Videos in Italian; specific case studies and stories for role-plays; real-time word cloud followed by group discussions; tasks related to the internship

^a^The students were divided into two groups, each attending 3 lessons

#### Measures and analysis

Pilots were evaluated by students and educators through a questionnaire developed within the project [see Additional file 3] and aiming to assess the integration of the IMPACCT training material into existing curricula. The four pilots represent three possible ways of integration: (1) a thematic course (optional), (2) additional lessons in a compulsory Laboratory, and (3) an optional extra activity. Pilot 2 was the same pilot type as 1, but online, therefore a completely different setting compared to pilot 3 and 4, making comparison difficult. Therefore, the evaluation through the questionnaire was only performed on Pilots 1, 3 and 4. At the end of each pilot, educators and students were asked to provide feedback on the lessons. The forms were anonymous and included open and closed questions aimed to assess the satisfaction of the participants and the relevance of the topic. Data were recorded in a devoted database to be analysed; open answers were transcribed in the original language and then translated for presentation in this paper.

In addition, each pilot assessed the students’ competence development using different techniques and tools according to the specific learning objectives defined and education activities carried out (for an example, see Table 2a of Supplementary material 2). Pilots 1 and 3 used group discussions elicited by real-time word clouds. Also, in Pilot 3 educators asked students to observe health professionals’ behaviours during their internship period, paying attention to the behaviours that more experienced health professionals ideally should have displayed (e.g. non-judgemental, respectful, empathic) [52]. Then, after one month they organised a meeting with students to analyse their observations. Pilot 4 proposed an additional pre-post-specific questionnaire, adapted from Mackert et al. [47] and Staufenbiel [48], to assess changes in HL awareness after the training. The questionnaire included 12 items representing aspects of health literacy, which students had to rate (from strongly disagree to strongly agree) on a 5-point Likert scale. The total score is calculated as the sum of items’ scores and ranges from 12 to 60. Cronbach’s alpha was 0.713 (n = 18) in the pre-test and 0.823 (n = 17) in the post-test. Finally, Pilot 2 used a written self-assessment test, consisting of 28 multiple-choice questions (MCQs), distributed at the end of each learning module. The students were asked to answer correctly each subgroup of MCQs to be allowed to progress through the course.

Data analysis was performed by integrating quantitative and qualitative results, which are reported in this way. Descriptive statistics were used to summarize the results of the evaluation questionnaires. Data are expressed as frequencies or mean (SD). Qualitative data were analysed manually summarizing the main aspects mentioned and extrapolating relevant quotations. Pre-post questionnaires were analysed through the Wilcoxon signed-ranks test and paired t-test; as the results of the two tests were equal, here we report paired the t-test and data expressed as mean (SD). Data analysis was performed with the statistical software Stata.

### COVID-19 pandemic online training

During the COVID-19 pandemic, three additional pilot tests (Pilots 5–7) were carried out in Italy and Germany, to evaluate the feasibility of the educational programme through online and hybrid learning, respectively. The lessons were organized by UNIVPM in Italy and Jacobs University (now named Constructor University) in Germany, during the period September to December 2020.

In Italy, two online courses were organized. Pilot 5 was an optional thematic course of 25 h for medical students. It used the material of Pilot 2 and was organized into 15-minute video lectures and self-assessment tests for each unit. Example activities included the viewing and commentary of video clips taken from films, exemplifying the perception of the sick person, as well as examples of doctor-to-patient communication being completely indifferent to the level of health literacy of the patient. Pilot 6 was organized as an optional thematic course for Nursing and Speech Therapy students. The course used the material of pilot 1, with a mixed didactic approach: asynchronous theoretical lectures on the definition and meaning of HL (two didactic units, 8 modules) and 6 video lectures displaying and commenting on clinical cases highlighting the impact of limited HL on the clinician/patient relationship (e.g. unexpected information requests, communication between health workers and relatives, communication misunderstandings, clinical cases and the teach-back technique). Students were also provided with in-depth material on the topics addressed during the course. Both courses used a self-assessment test consisting of MCQs to guide learning progressions through the different modules. Evaluation of the two online courses was done through a group discussion at the end of the lessons.

In Germany, Pilot 7 was organized as one hybrid course (seminar and lab). This was a mandatory course for bachelors’ students studying psychology. It included 28 sessions of 75 min and addressed, in addition to the topics mentioned above with the course in Italy, (1) health psychology, (2) theories and models of health behaviour change, (3) health, well-being and coping with stress; (4) communication techniques and (5) designing and implementing interventions. The course was hybrid, organized at the beginning with some students being in the classroom and those in quarantine or not able to travel to campus participating online and then pivoted to completely online. The evaluation of this course was done through an evaluation procedure after the last lesson.

## Results

### Evaluation of the educational programme

One hundred and seven students participated in the Pilot tests 1–4 in Italy, of which 74 were females and 33 males (Table 3). The courses were delivered by 6 educators, 5 females and 1 male.

**Table 3:** Pilot tests participants

	Pilot 1 (Nursing and Speech Therapy)	Pilot 2 (Medicine and Surgery)	Pilot 3 (Physiotherapy)	Pilot 4 (Midwifery)
Students				
N (Male/Female)	36 (7/29)	22 (10/12)	32 (16/16)	17 (0/17)
Age range (years)	21–42	23–27	19–36	20–24
Educators				
N (Male/Female)	2 (1/1)	1 (0/1)	2 (0/2)	2 (0/2)

### Students evaluation

#### Overall experience

**Fig. 2:**
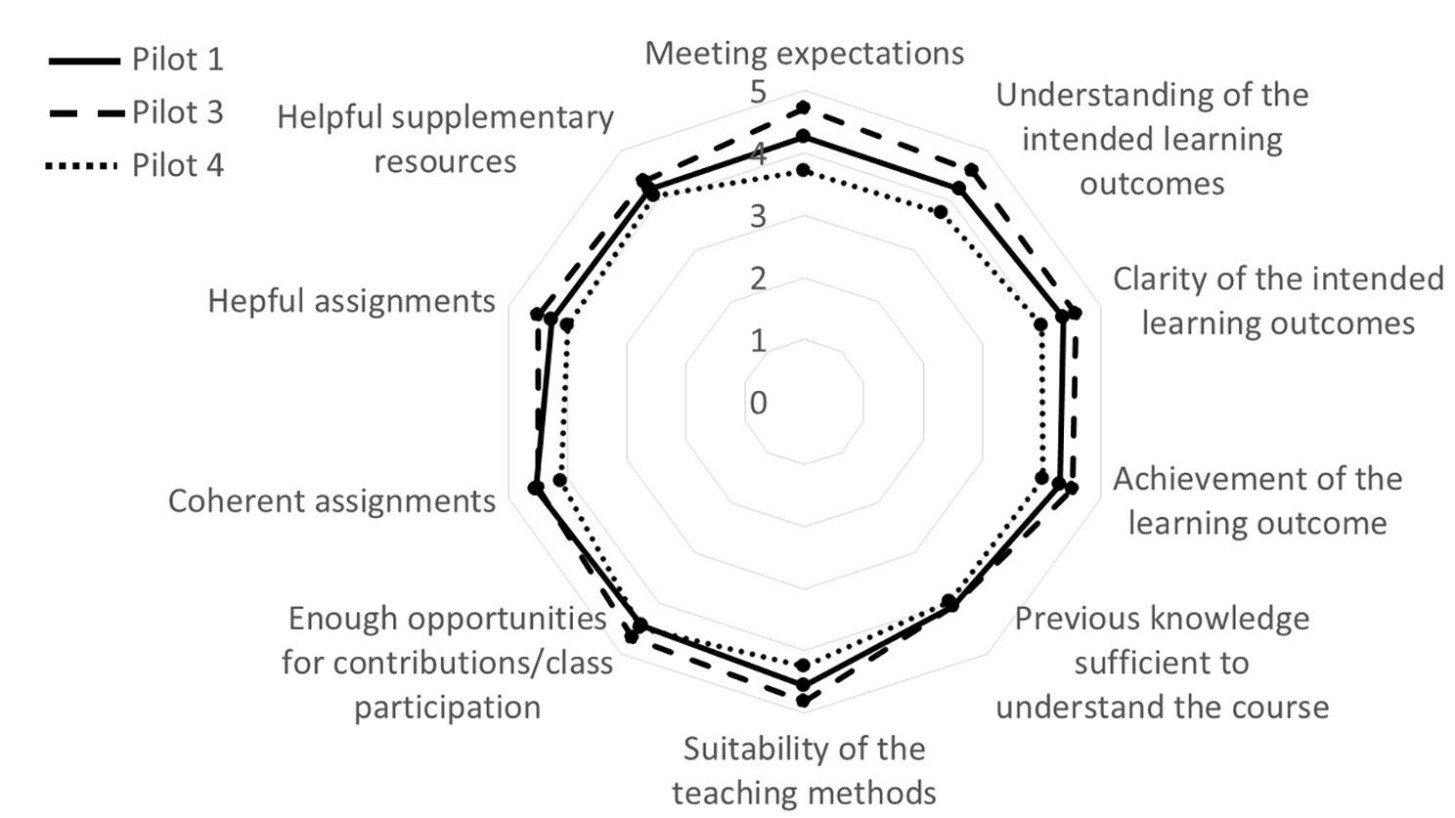
Students’ evaluation (values ranging from 1 “Completely disagree” to 5 “Completely agree”)

Eighty-five students answered the evaluation questionnaire, of which 62 females and 23 males aged between 19 and 42 years (Pilots 1, 3 and 4). Overall, the students gave positive feedback to the lessons attended (Fig. 2), considering them clear and useful, carried out with appropriate methods and providing sufficient opportunities for discussion. Overall, the experiences were aligned with the expectations of the students. Almost all students stated their interest (96%) and satisfaction with the courses (99%).

#### Key elements

Eighty-three out of 85 students would recommend the courses to other colleagues. Table 4 shows some relevant quotations from the questionnaires. The main reasons provided were: (i) the lessons are useful/essential for the traineeship and the (future) profession (n = 21); (ii) they allow understanding of important aspects of healthcare professional-patient relation (n = 20); (iii) the teaching methods used (n = 14); (iv) the focus on aspects that are often taken for granted and not addressed in other courses (n = 12); (v) the importance of patient communication (n = 12).

**Table 4:** Students’ comments (from evaluation questionnaires; pilot number in brackets)

- I recommend these lessons because…
o … they deal with topics that may seem obvious and one does not always think about it, but they are very important (P3). o …allow us to know and understand the critical issues that can be encountered in the health care sector about the relation with the patient and how to deal with them in the appropriate way (P1). o … it is essential that healthcare professionals help patients to understand what they said both by showing empathy and by giving explanations. (P4).
- This course is an opportunity that is given to us in a sea of notions to let us know how to do things and few to let us know how to be (P3). - It was very significant to be able to explain theoretical concepts through sympathetic examples that remain in the mind (e.g. alien and folded sheet of paper exercises) (P3). - Role playing has surely helped to learn and deepen the knowledge (P1). - I appreciated the importance that has been given to communication in general and how “the other’s” responses can change according to our attitude, which is the basis of everything (P1).

Participants particularly appreciated the teaching methods - such as the examples of patient communication (e.g. active listening, teach-back technique, non-verbal communication, behaviour modification), the practical exercises (e.g. role-playing and open discussions), and the videos, evaluating them as applicable to their practice. The theoretical parts on health literacy and some already known concepts were considered less interesting, being mentioned by only 20 students (23%). Finally, students provided the following suggestions for improvement (from the most to the least mentioned, multiple answers possible): provision of the teaching materials in advance (n = 24; 28.2%), improvement of coordination with the contents of other courses (n = 23; 27%), increase of the quality of teaching materials (n = 19; 22.4%), removal of topics already contained in other courses (n = 18; 21.2%), and provision of more basic knowledge (n = 11; 12.9%). Some students reported their concerns about the ability to implement the strategies and techniques learned in their work, asking for more opportunities to experience them in class.

#### Assessment of the students’ competence development

The additional evaluation activities provided insights on students’ behavioural changes after the courses (Pilots 1 and 3) and achievement of the learning outcomes (Pilots 2 and 4). Pilots 1 and 3 used a real-time word cloud exercise to ask students to represent the main important behavioural aspects of their work and the behaviours observed in other health professionals during their internship experience, respectively. The aspects mentioned more frequently in both Pilots were: attitudes (being calm, kind, available, empathic, confident, professional, patient, positive, precise, equal, tolerant, participative, and being able to adapt to the environment and the patients); interactions with the patients (i.e. humanity, focus on receiving the patients adequately, listening and observing them actively and carefully, be attentive, communicating clearly and checking understanding, reassuring and encouraging them, respecting their needs, and promoting a good interaction); and relation with others (i.e. the importance of collaborating and sharing information with both other healthcare professionals and patients’ family members). Students of Pilot 3 showed a good understanding and a great ability to internalize key concepts that emerged, and were able to recognize good examples of communication in the context of health literacy between health professionals and patients.

In Pilot 2, the written self-assessment test resulted in all participants achieving the learning outcomes, passing the exam on their first trial. In Pilot 4, the pre-post assessment on HL awareness (Table 5) was completed by 14 out of 17 students. Overall, there was an improvement in knowledge of HL (p < 0.001), with a mean total score passing from 46.5 (SD = 4.13; range 40–55) to 52.71 (SD = 3.99; range 46–59).

**Table 5:** Pre-post self-evaluation of health literacy knowledge (Pilot 4, n = 14)

Item	Pre Mean (SD)	Post Mean (SD)	p-value
1. I am aware of the meaning of the term health literacy	3.57 (0.65)	4.79 (0.43)	**< 0.001**
2. I am aware of what it means for patients to have low health literacy	3.64 (0.93)	4.5 (0.52)	**0.017**
3. It is important to educate patients with limited health literacy to perform adequate self-management behaviour	4.07 (0.47)	4.64 (0.5)	**0.001**
4. I know what active listening is and if so, I use active listening to gather information	3.71 (1.07)	4.43 (0.51)	0.055
5. I can easily interpret cues related to non-verbal communication of patients	3.43 (0.76)	4.07 (0.62)	**0.022**
6. I can understand and have patience with others’ beliefs and values	4.07 (0.47)	4.21 (0.58)	0.435
7. It is important to explore and respond to patients’ emotions’	4.36 (0.63)	4.57 (0.65)	0.272
8. It is important to limit the amount of information you provide to a patient	2.93 (1)	3.36 (1.08)	0.272
9. It is important to repeat information to patients	4.5 (0.65)	4.64 (0.5)	0.435
10. It is important to draw pictures in addition to providing verbal information for patients	3.57 (0.76)	4.64 (0.5)	**< 0.001**
11. It is important to encourage people to ask questions at different times during an interaction	4.36 (0.63)	4.64 (0.5)	0.104
12. It is important to involve the preferences of the patient with regard to treatment	4.29 (0.73)	4.21 (0.7)	0.720
Total	46.5 (4.13)	52.71 (3.99)	**< 0.001**

Note: Scores range from 1 (completely disagree) to 5 (completely agree)

After the training, students significantly improved their awareness of the meaning of HL (item 1; p < 0.001), its impact on patients (item 2; p = 0.017), the importance of educating patients in regard to self-management (item 3; p = 0.001), non-verbal communication (item 5; 0.022), and the use of pictures (item 10; p < 0.001). It is worth noting that, although the meaning of HL was not perfectly clear before the start of the lessons, some related concepts were already known and did not show significant improvement after the lessons (p > 0.05), (e.g. the importance of patients’ beliefs and values (item 6), emotions (item 7), engagement (item 11) and preferences (item 12), as well as the importance to repeat information to patients). Active listening (item 4) showed a slight improvement, even if not statistically significant (p = 0.055). A possible explanation is that this item relates both to understanding and acting properly, and students could feel confident about their understanding but were not yet able to put theory into practice. The lowest score was reported in item 8 “It is important to limit the amount of information provided to a patient”, which did not show an improvement after the course and needs to be better addressed in future lessons.

### Educators evaluation

Educators were asked to evaluate the adequacy of the training material, its possible integration into the existing curricula, and any organizational problems. Overall, the feedback received was positive. The didactic material, as well as the previous knowledge of the educators, were considered sufficient to carry out the programme. The teaching methods and the proposed activities were evaluated as adequate. Three out of six educators had no clear opinion (i.e. neither agree nor disagree) about the clarity of learning outcomes. Table 6 shows some relevant quotations from the questionnaires.

**Table 6:** Educators’ comments (from evaluation questionnaire)

- Practical exercises have allowed for more settled experiential learning (applied to the traineeship).
- The less valuable part of the course was the introductory part, that could be shortened
- It was important to put communication and the relationship between practitioners and between practitioners and patients at the centre of an educational path, and to be able to link these elements to the practical traineeship experience.
- These lessons can be linked to other content by integrating them into the first year’s teaching modules to lay the foundations for future course years.

Educators’ answers on the validity of the elements of the courses were in line with those of the students. They appreciated the material on the diversity topic, the different aspects of communication, and the interactive activities (e.g. exercises and role-playing activities). However, they defined the introductory part as less interesting and noted some repetitions which could be avoided.

Educators confirmed that the content of the lessons is easily transferable into practice, highlighting the need to promote a more efficient coordination with other courses. Although all educators stated that the courses took place as planned, when asked if they could change anything, what that may be, they reported: improving the integration of the materials with contextualized information, including additional practical activities and real scenarios on video in students’ native language, and focusing more on the relationship among health professionals.

The practical exercises, role-playing activities and group discussions were considered useful tools to evaluate students’ competence development. A very positive evaluation was given to the pre-post questionnaire used in Pilot 4.

Finally, educators were asked how they would use the material in their future courses. They confirmed the validity of the integration options proposed in the pilots and proposed additional solutions, such as identifying learning outcomes (and related training material) linked to the internship and integrating the discussion of clinical cases into additional relevant aspects, e.g. shared decision-making and the role of caregivers. Moreover, it was proposed to include small modules in each academic year to develop a specific training pathway on HL, from basic to advanced skills.

### Online training during the COVID-19 pandemic

In Italy, two online courses were implemented. Pilot 5 was an optional thematic course (8 modules) for medical students, which was attended by 69 students (37 females and 32 males), aged between 23 and 25 years. A significant increase in the number of participants was observed compared to the previous year, likely due to the positive feedback received by the students from their peers. The inclusion of videos of patients reporting their point of view was one of the most appreciated components of the course. The online format impeded the realization of some learning strategies like role-playing; therefore, other approaches were used (e.g. listening to interviews) to elicit changes in the beliefs and attitudes of the students towards the patients’ health literacy. The second (Pilot 6), an optional thematic course for Nursing and Speech Therapy, was followed by 20 students (18 females and 2 males) between 21 and 32 years old. The students were highly satisfied with the course and appreciated the transition from the live course to the online course. A mixed didactic approach was followed (asynchronous theoretical lectures and video lectures) and a self-assessment test had to be completed at the end of each module to unlock access to the next one. All the participants were successful in the achievement of the learning outcomes.

In Germany, Pilot 7 was carried out as a bachelor course conducted in a hybrid form (online and in presence) and attended by 39 students aged 18 to 21 years. Students rated evaluation items with a mean level of 2 (good) out of a scale from 5 (bad) to 1 (very good). Open feedback is presented in Table 7. The professor evaluated the course and the learning material as good and no concrete requirements for adaptations were deemed necessary.

**Table 7:** Feedback on the hybrid course (Pilot 7, Germany)

- “Made the course interactive by asking different students each time to interact/answer questions. Taught wide array of things”
- “The instructor made the “lab” section of the two classes very interactive to reinforce what we learned. Structured syllabus and lesson plan, informative slides, and engaging class were all great.”
- “The online teaching went well in this course due to the effective methods used by the instructor, such as different kinds of quiz related to the learning topics”
- “I would suggest to give students more time on tasks. I felt rushed sometimes. The online format is difficult but we managed well.”
- “This was my favourite class, I really enjoyed to take part of it and Prof. make it really dynamic. Keep it like this! :-)”
- “I thought it was really impressive how easily the professor switched to online format and still was able to structure the course interestingly.”

## Discussion and conclusions

This study described the development and evaluation of an evidence-based HL educational programme for health professional undergraduate students based on an iterative co-creation process. It also described how it was used during times of crisis such as the COVID-19 pandemic with travel restrictions and quarantine regulations. Evaluation of the educational programme received positive feedback from students and educators. Moreover, the evaluation showed the feasibility of the educational programme with its high flexibility in fitting different courses and disciplines and online delivery in response to the COVID-19 pandemic.

The educational programme was based on existing theories, models and educational frameworks and informed by a systematic review and literature search on needs, experiences and preferences of patients as well as on main intervention strategies to enhance health literacy outcomes for people with limited health literacy.

The educational programme was perceived as relevant and useful by both students and educators. They recognized the relevance of the topic and were satisfied with the organization and contents of the courses. Furthermore, educators confirmed the high flexibility of the educational programme in fitting into mandatory or voluntary, long or short courses in a wide range of disciplines in multiple jurisdictions. An important explanation for these positive results might be the strong involvement of relevant stakeholders in the co-creation process allowing the development of an educational programme tailored to their needs and perspectives.

The co-creation with multiple stakeholders has influenced the content, learning activities and approach of the educational programme. The training material includes basic concepts of HL (e.g., definition, prevalence, impact) as well as advanced aspects (e.g., hospital discharge, healthy behaviours, eHealth), which can be addressed singularly, or integrated into other specific health topics. We called this approach the ‘supermarket model’ because educators can choose and mix topics and activities based on their needs. Previous studies integrated HL education through dedicated workshops or as part of other courses, and applied a classroom-based approach with interactive activities such as discussions, role-play, and case studies [26, 49]. IMPACCT used the same approach, testing different options for integrating training material into the existing curricula. Previous experiences of HL training showed increased competencies and behaviour changes [30–34]. Similar results were observed in the evaluation of this educational programme.

Could this educational programme contribute to patient-centred care? We cannot answer this question based on our evaluation but students reported the importance of the topic for their profession and understood the impact of their behaviours on the relationship with patients and in the care process as a whole. The most cited aspects to improve and apply in their future work were empathy, active listening, observation, and patience [50]. It is particularly relevant that students of all pilots underlined the importance of active listening and taking into account patients’ needs, beliefs and preferences. Patient-centred care is associated with better health outcomes and it is essential for patients with limited HL, who have difficulties in managing their health [14]. In line with other studies, the pre-post evaluation carried out in Pilot 4 confirmed a significant improvement in the awareness of the term HL [30, 51].

The pilots carried out during the COVID-19 pandemic showed that the training material is also adaptable to online delivery and hybrid training. Although some activities cannot be performed remotely, students reported positive feedback and found the alternative activities proposed useful, such as the viewing and discussion of videos about patient-professional communication. Some of the activities could be further developed beyond the Covid-19 emergency period to support traditional training, for example providing students with basic knowledge on the topic of HL before starting lessons, or used as supplementary material for those interested.

The main strengths of this study are the co-creation of the programme (e.g. [22]). with a large group of stakeholders and the evaluation of both students and educators. This study presents a few limitations. The methods of stakeholders’ recruitment and pilot settings and students involved could have produced some (self-selection) bias in the study. The pilot design did not allow a control group to account for confounding factors and the small size of the group performing the pre-post evaluation limits the generalization of the results. As explained by Velthius et al. [52], the process of curriculum change can be addressed through different approaches and strategies. In this study, the pilot tests were used to allow educators to apply and evaluate the training material to support its later integration into the standard training offering.

Society as a whole is increasingly recognising the urgent need to improve access to healthcare information and services, to achieve better health outcomes. The COVID-19 pandemic has highlighted even more the importance of HL. Health literacy education is a useful tool to address this challenge while improving the sustainability of healthcare systems. Furthermore, the training material is a suitable tool to support online teaching during the COVID-19 pandemic which could be further exploited after the end of the emergency. There is a large and discernible gap in HL education in the undergraduate health professional curriculum. There is also the need to provide more accurate studies on evaluation of HL educational programmes. Integration and sustainability of HL education requires capacity building and a flexible approach to meet the learning needs of students and teaching needs of educators in the diverse European education and health system environment. Programmes like IMPACCT can contribute to meeting these needs and in this way, existing inequalities can be addressed and health systems move towards improving patients’ and citizens’ health outcomes.

### Electronic supplementary material

Below is the link to the electronic supplementary material.

**Additional file 1:** Co-creation process with multiple stakeholders. Summary table of stakeholders’ consultations outcomes.

**Additional file 2:** Educational material. Examples of a navigation template (Supplementary Table 1), an educational activity (Supplementary Table 2), and the overview of the educational programme (Supplementary Table 3).

**Additional file 3:** Evaluation questionnaire.

## Data Availability

The datasets used and analysed during the current study are available from the corresponding author upon reasonable request.

## List of abbreviations

HL: Health Literacy
LUs: Learning Units
UNIVPM: Polytechnic University of Marche
MCQs: Multiple choice questions
SD: Standard Deviation
