# Supplementary material for: Health literacy education at the time of COVID-19: development and piloting of an educational programme for university health professional students in 4 European countries
Source: BMC Med Educ. 2023 Sep 8;23:650. doi: 10.1186/s12909-023-04608-3 (PMC10492329; doi:10.1186/s12909-023-04608-3)
Supplement: Supplementary file 1 — Additional file 1: Co-creation process with multiple stakeholders. Summary table of stakeholders’ consultations outcomes. [file 12909_2023_4608_MOESM1_ESM.pdf]

Additional file 1: Co-creation process with multiple stakeholders.

Pha-se	Event & participants	Objective	Outcomes of the consultations	Implementation
1	Kick Off meeting, UMCG (Netherlands), October 2017; Project partners*, healthcare professionals, education experts	Define educational philosophy and core components of the educational programme (EP)	– Agreed definition of health literacy<br>– Initial definition of topic areas<br>– Recognition of pressure on healthcare professional curriculum<br>– Active inclusion of students in early stages of training (with/without patient contact)<br>– Outcome measures developed considering all target groups<br>– Importance of including diversity into the EP (e.g. background of the learners, educational systems, communication to different contexts and cultures)<br>– Importance of coherence both within and between learning units in terms of outcomes, content, teaching strategies, assessment, and program objectives<br>– Perspectives of stakeholders on the change processes in different educational settings in Europe	– Consistent use of shared understanding of health literacy<br>– Topic areas further developed in phase 2<br>– Pilots with various student groups<br>– Inclusion of diversity as a transversal aspect of the programme<br>– Flexible and adaptable design of the programme<br>– Development template to facilitate coherence<br>– Research with educators to explore change undertaken (48)
	Learning Event, UMCG (Netherlands), February 2018; Project partners*, health professional education experts	Identify stakeholders' needs, perspectives and preferences	– Aspects to be included in the educational programme: identification of health literacy problems; patient-provider interaction barriers (e.g. posing questions); patients' preferences (e.g. culture, education, experiences); mental health literacy (e.g. co-occurrence of chronic diseases with mental health problems, coping with bad news).<br>– Use of newer definition of health by Huber et al. (49) with more emphasis towards the ability to adapt and self-manage in face of social, physical, emotional challenges.<br>– Take into account the future implementation of the EP in other settings (e.g. promoting sustainability).<br>– Take into account students' prior knowledge, skills or motivation<br>– Using an iterative process for the development of learning units	– Inclusion of the mentioned aspects in the programme<br>– Adaptability and self-management issues considered in the development of the programme<br>– Different settings explored<br>– Students involved in the co-creation process<br>– Iterative process applied for the development of the programme involving multiple stakeholders
2	Transnational Meeting, Pavol Jozef Šafárik University of Košice (Slovakia), June 2018;	Improve the content of the learning units and	– Health Literacy education confirmed as valuable<br>– Need to strengthen communication, leadership, and advocacy competences in healthcare professionals<br>– Need to harmonize education practices in health literacy across Europe	– Learning unit content adapted to identified needs through an iterative process

	Project partners*, students, communication and HL educators, course coordinators, healthcare professionals, representatives of patient organisations	evaluate strategies for uptake of the educational programme	– "Supermarket model" approach to facilitate the integration of health literacy education in different curricula<br>– Promotion of evidence-based educational strategies with problem-and practice-based learning to create realistic educational activities<br>– Use of theoretical frameworks or models to ensure coherence<br>– Stakeholders as agents for change	– Development of 'supermarket model' promoting flexibility and adaptability in use of learning materials<br>– Examples of materials use included in programme manual<br>– Best practices and inspiring examples included in the programme<br>– Continued co-creation with stakeholders
3	Transnational meeting, Jacobs University (Germany), September 2019; Project partners*, educators, researchers, healthcare professional, students	Identify actions, policies, and strategies for the introduction of Health Literacy in higher education	– Adding interactive content in the courses, to help integrating the demands of students with different background<br>– Sensitize a larger group of people of the importance of HL at all levels, also through bottom-up and peer-to-peer approaches<br>– Propose educators and organizations awards in the field of health literacy<br>– Continuity in health literacy education programs with effective evaluation tools for teaching and learning, outcome performance and practices.<br>– Continuous evaluation and improvement of HL education beyond the project<br>– Connect to institutions who have successfully implemented HL education to present their success stories as hard evidence<br>– Start including HL as early as possible (e.g. preschool age)<br>– Present evidence/effectiveness/patient perspectives/cost & quality of patient safety<br>– Develop policies and plans for the implementation of the HL program	– HL programme and MOOC developed according to feedbacks and available online<br>– Policy brief developed according to feedback and disseminated<br>– Programme manual developed<br>– Challenges identified and addressed including: participation of students, measuring and increasing impact of project, influencing key stakeholders and branding strategy, coherence of the educational programme, sustainability (access & legal issues), patient-centeredness.<br>– IMPACCT recognised as a good practice by the European Thematic Network 'Profiling and training the health care workers of the future' in 2020

Note: * University Medical Center Groningen (UMCG), University of Groningen (Netherlands); National University of Ireland Galway (Ireland); Jacobs University (Germany); Marche Region – Regional Health Agency (Italy); Pavol Jozef Šafárik University of Košice (Slovakia).
