# Supplementary material for: Health literacy education at the time of COVID-19: development and piloting of an educational programme for university health professional students in 4 European countries
Source: BMC Med Educ. 2023 Sep 8;23:650. doi: 10.1186/s12909-023-04608-3 (PMC10492329; doi:10.1186/s12909-023-04608-3)
Supplement: Supplementary file 2 — Additional file 2: Educational material. Examples of a navigation template (Supplementary Table 1), an educational activity (Supplementary Table 2), and the overview of the educational programme (Supplementary Table 3). [file 12909_2023_4608_MOESM2_ESM.pdf]

Additional file 2: Educational material

Supplementary Table 1: Example of a Navigation Template from the Advanced Learning Unit Shared Decision Making

**Learning Outcomes:**<br>The student is able to…	**Educational Activities**	**Material Available**	**Assessment**
… explain the difference between decision-making styles.	Introductory lecture<br>EA1: small group work, student exercises	• SDM _Introduction.pptx<br>• SDM_EA1_exercises.docx<br>• SDM_EA1_explanations.docx	EA1: Group reflection and discussion
… describe the key components of SDM	Introductory lecture<br>EA1: small group work, student exercises	• SDM _Introduction.pptx<br>• SDM_EA1_exercises.docx<br>• SDM_EA1_explanations.docx	EA1: Group reflection and discussion
... recognize the value of SDM in healthcare encounters	Introductory lecture<br>EA1: small group work, student exercises	• SDM_ Introduction.pptx<br>• SDM_EA1_exercises.docx<br>• SDM_EA1_explanations.docx	EA1: Group reflection and discussion
… use strategies to integrate SDM in daily practice	EA3: role play, small group work	• SDM_P2_SDMinpractice.pptx<br>• SDM_EA2_Roleplay.docx	EA3: Feedback from peers or educator(s) based on observation + group reflection and discussion
… express their attitudes regarding the value of SDM	EA2: individual assignment	• SDM_EA3_blog.docx	EA2: Educator and/or peer review

SDM is Shared Decision Making, denoting the learning unit the material belongs to

EA Educational Activity, links to and cross references with other materials within the learning unit

Supplementary Table 2: Example of an Educational Activity from the Learning Unit Health Literacy Challenges.

Activities are supported by information for educators.

Below you see three parts of conversations between doctors/nurses and patients diagnosed with Type 1 Diabetes. Read through these fragments and indicate how much you think the patients understood what the doctor/nurse told them.

Example 1:

01	Doctor:	So how often do you test your blood sugar?
02	Patient:	Uh two times a day?
03	Doctor:	Yes
04	Patient:	Oh
05	Doctor:	Alright, so you can make an appointment to see me again in six months

A. How would you rate the level of understanding of this patient? Provide a rating between 1 and 5, where 1 means 'no understanding' and 5 'full understanding'.

Example 2:

01	Nurse:	To find out what your blood sugar level is at that moment in time you need a blood testing meter, a finger prick device, some test strips and a lancet *((reads))*
02	Nurse:	Do you know what a lancet is?
03	Patient:	Yes that's this thing right? *((points to lancet on the table))*

B. How would you rate the level of understanding of this patient? Provide a rating between 1 and 5, where 1 means 'no understanding' and 5 'full understanding'..

Example 3:

01	Nurse:	So I will explain it to you once more. When you wake up and before you've eaten, your blood sugar level should be between 5 to 7mmol/l. Now you do understand?
02	Patient:	Yes
03	Nurse:	Okay

A. How would you rate the level of understanding of this patient? Provide a rating between 1 and 5, where 1 means 'no understanding' and 5 'full understanding'.

Compare your scores to those of your fellow students. Now that you have seen how the levels of patient understanding were perceived by you and the other students, please have a look at the three parts of conversations again. Why did you rate some examples higher than others? Please discuss with other students.

Supplementary Table 3: Overview of the Education Programme

Theme	Learning Unit
**The basics**	Introducing health literacy
	Health literacy skills
	Working with diverse populations
**Strengthening social supports**	Patients, families and support groups
**Strengthening self-management**	Promoting health behaviours and health literacy
	E-health literacy
	Food literacy
	Mental health literacy
	Chronic disease self-management
**Improving healthcare professionals' interpersonal skills**	Health literacy challenges
	Shared decision making
	Tailoring interpersonal communication
**Overcoming organisational barriers**	Organisational health literacy
	Patient discharge and rehabilitation
	Patient safety and quality care
