# Supplementary material for: Health literacy education at the time of COVID-19: development and piloting of an educational programme for university health professional students in 4 European countries
Source: BMC Med Educ. 2023 Sep 8;23:650. doi: 10.1186/s12909-023-04608-3 (PMC10492329; doi:10.1186/s12909-023-04608-3)
Supplement: Supplementary file 3 — Additional file 3: Evaluation questionnaire. [file 12909_2023_4608_MOESM3_ESM.pdf]

Additional file 3: Evaluation questionnaires

Instruction for using this evaluation form:

Pls print out the appropriate pages you find in the following and hand them out to the participant of project: there are pages for students and pages for educators, and for both.

Pls add your own contact details to the 2nd page so that the participants know whom to contact in case of questions or further feedback.

ABOUT THIS QUESTIONNAIRE

This questionnaire is a part of the IMPACCT project. We greatly value your past participation in the project, and we hope that you will find this evaluation form interesting to complete. As always, your answers are extremely important to us. Please remember that your participation is voluntary and that you may skip over any questions that you would prefer not to answer. By answering the following questions you provide us the permission to use your data anonymized. The IMPACCT team covers this research in order to help ensure your privacy.

Many questions can be answered by placing an (X) in the box to indicate your response. Some questions may not apply to you, and you will be instructed to skip them. When this occurs, you will find an arrow (→) from your answer to the next appropriate question number. When no special instruction is given for your response choice, please continue with the next question.

If you have any questions about the evaluation for, please feel free to as the project member who handed you this form or write us an email.

THANK YOU!

Your IMPACCT-Team

FOR STUDENTS

1. **Please think about the course you just participated in and the educational process i.e. what you experienced.**

Criteria	Rating					
	1 = Completely disagree	2 = Disagree	3 = Neither agree nor disagree	4 = Agree	5 = Completely agree	NA
1.1. The course met my expectations	1	2	3	4	5	NA
1.2. I understood and still remember the intended learning outcomes	1	2	3	4	5	NA
1.3. The intended learning outcomes were clear	1	2	3	4	5	NA
1.4. All/most of the learning outcome were achieved	1	2	3	4	5	NA
1.5. My previous knowledge in relation to the topics was sufficient to understand the course	1	2	3	4	5	NA
1.6. For me, the teaching methods and activities were suitable for the achievement of the learning outcomes	1	2	3	4	5	NA
1.7. There were sufficient opportunities for me for contributions/class participation	1	2	3	4	5	NA
1.8. Assignments were related to the expected learning outcomes	1	2	3	4	5	NA
1.9. Assignments helped me to meet expected learning outcomes	1	2	3	4	5	NA
1.10. Supplementary resources were helpful in meeting the expected learning outcomes	1	2	3	4	5	NA

ADDITIONAL ITEMS

2. **Are you interested in the subject taught during the course?**

Absolutely uninterested	Rather uninterested	Fairly interested	Absolutely interested
☐	☐	☐	☐

3. **Overall, how satisfied are you with this course?**

Absolutely unsatisfied	Rather unsatisfied	Fairly satisfied	Absolutely satisfied
☐	☐	☐	☐

4. **Would you recommend this course to other students?** ☐ **Yes** ☐ **No**

4.1. If yes, why?

____

4.2. If no, why?

____

5. **In your opinion, what were most valuable elements of this course?**

____

6. **And the worst?**

____

7. **In your opinion, what did you find most applicable for practice from the lesson?**

8. **Suggestions for improvements (tick all that you find appropriate)**

8.1. ☐ Reduce the overall course content
8.2. ☐ Provide for more basic knowledge
8.3. ☐ Delete from the programme topics that are already contained in other courses
8.4. ☐ Improve the coordination with the contents of other courses
8.5. ☐ Increase the quality of the teaching material
8.6. ☐ Provide the teaching material in advance
8.7. ☐ Other :____ (*please specify*)

Open-ended questions for students and facilitators:

____

9. **Other comments?**

____

Some more questions:

Are you a ...

- ☐ student
- ☐ doctor
- ☐ nurse
- ☐ teacher, health educational professional
- ☐ researcher
- ☐ other (please specify):

Are you …

- ☐ Female
- ☐ Male
- ☐ Other

How old are you? ______

In which country are you mainly working? ______

FOR EDUCATORS:

1. **Please think about the course you just taught and the educational process i.e. what you experienced.**
   1.1. **The educational material was sufficient to carry out the course**

1 = Completely disagree	2 = Disagree	3 = Neither agree nor disagree	4 = Agree	5 = Completely agree
☐	☐	☐	☐	☐

Criteria	Rating					
	1 = Completely disagree	2 = Disagree	3 = Neither agree nor disagree	4 = Agree	5 = Completely agree	NA
**1.2. The course met my expectations**	1	2	3	4	5	NA
**1.3. I understood and still remember the intended learning outcomes**	1	2	3	4	5	NA
**1.4. The intended learning outcomes were clear**	1	2	3	4	5	NA
**1.5. All/most of the learning outcome were achieved**	1	2	3	4	5	NA
**1.6. My previous knowledge in relation to the topics was sufficient to understand the course**	1	2	3	4	5	NA
**1.7. For me, the teaching methods and activities were suitable for the achievement of the learning outcomes in my students**	1	2	3	4	5	NA
**1.8. There were sufficient opportunities for me to enable for contributions/class participation**	1	2	3	4	5	NA
**1.9. Provided assignments were related to the expected learning outcomes**	1	2	3	4	5	NA
**1.10. Provided assignments helped me to meet expected learning outcomes**	1	2	3	4	5	NA
**1.11. Provided supplementary resources were helpful in meeting the expected learning outcomes**	1	2	3	4	5	NA

2. **Did everything go according to plan during the lesson? Yes☐ No ☐**

(If not, please explain)

____

If you could change anything, what would you have done differently?

____

How did you assess student learning as you went through the lesson? What did this tell you? Is there anything that you need to spend some additional time on based on the feedback received from these assessments?

____

How would you utilize what you taught today to make connections with previously taught content as well as future content?

____

FOR STUDENTS AND FACILITATORS:

10. In your opinion, what were most valuable elements of this course?

____

11. And the worst?

____

12. What would you recommend to improve the course?

____

13. Other comments?

____

Suggestions for improvements (tick all that you find appropriate)

- ☐ Reduce the overall course content
- ☐ Provide for more basic knowledge
- ☐ Delete from the programme topics that are already contained in other courses
- ☐ Improve the coordination with the contents of other courses
- ☐ Increase the quality of the teaching material
- ☐ Provide the teaching material in advance
- ☐ Other: ____ (*please specify*)

Open-ended questions for students and facilitators:

14. In your opinion, what did you find most applicable for practice from the lesson?

____

15. Other comments?

____

Some more questions:

Are you a ...

- ☐ student
- ☐ doctor
- ☐ nurse
- ☐ teacher, health educational professional
- ☐ researcher
- ☐ other (please specify):

Are you …

- ☐ Female
- ☐ Male
- ☐ Other

How old are you? ☐

In which country are you mainly working? ☐
